# Supplementary material for: Effect of the Absence of α Carbonic Anhydrase 2 on the PSII Light-Harvesting Complex Size in Arabidopsis thaliana
Source: Plants (Basel). 2025 May 20;14(10):1529. doi: 10.3390/plants14101529 (PMC12114823; doi:10.3390/plants14101529)
Supplement: Supplementary file 1 [file plants-14-01529-s001.zip › plants-3627095-supplementary.pdf]

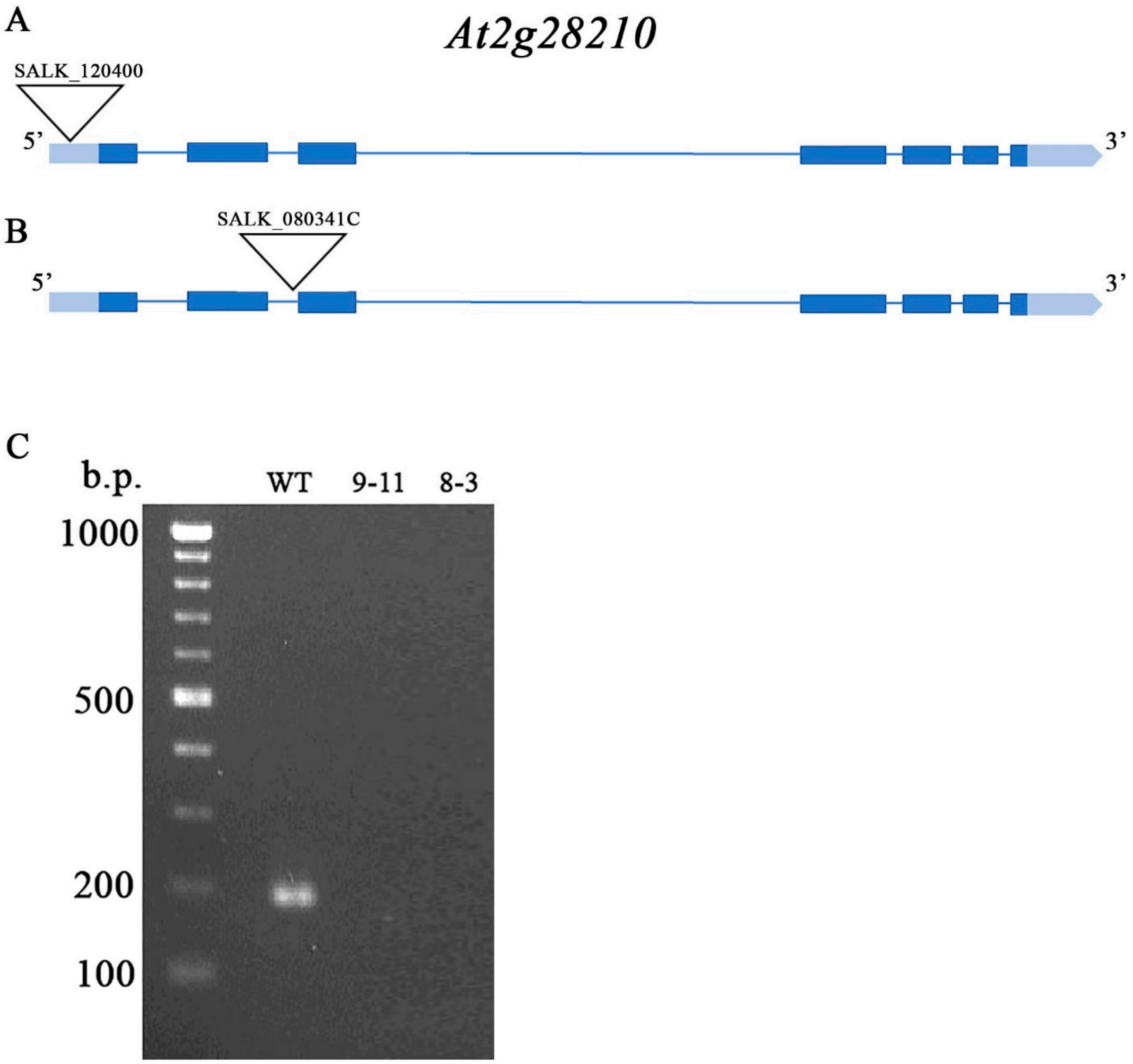

Supplementary Figure S1. Schematic representation of two T-DNA insertions in the *At2g28210* gene encoding α-CA2. SALK_120400 is the position of the insertion in homozygous mutant plants "9-11" line (**A**) and SALK_080341C is the position of the insertion in homozygous mutant plants, "8-3" line **(B**). Blue boxes represent exons (light blue areas are untranslated regions, dark blue boxes are the coding sequences) and blue lines indicate introns. The triangles indicate T-DNA insertions. **C** is the result of electrophoresis of PCR products obtained with specific primer pair of the *At2g28210* gene. cDNA synthesis was performed with RNA from WT plants, mutant line "9-11", mutant line "8-3". DNA ladder with DNA fragments ranging from 100 bp to 1,000 bp (Biolabmix, Novosibirsk, Russia) was used as a marker for electrophoresis.
